# Telehealth and In-Person Mental Health Service Utilization and Spending, 2019 to 2022

**DOI:** 10.1001/jamahealthforum.2023.2645

**Published:** 2023-08-25

**Authors:** Jonathan H. Cantor, Ryan K. McBain, Pen-Che Ho, Dena M. Bravata, Christopher Whaley

**Affiliations:** 1 RAND Corporation, Santa Monica, California; 2 RAND Corporation, Arlington, Virginia; 3 Castlight Health, San Francisco, California; 4 Brown University School of Public Health, Providence, Rhode Island

## Abstract

This cohort study assesses trends in monthly telehealth vs in-person utilization and spending rates for mental health services among commercially insured US adults before and during the COVID-19 pandemic.

## Introduction

Telehealth service utilization expanded rapidly at the COVID-19 pandemic outset, particularly for mental health conditions.^1^ Unlike physical health conditions that may require physical examinations or laboratory testing, many mental health services can be provided virtually.^2^ Three years after the 2020 SARS-CoV-2 national public health emergency (PHE) declaration, many facets of the US health care system have returned to normal.^3^ However, trends in mental health service utilization and spending before expiration of the PHE in May 2023 are largely undocumented. Therefore, we assessed monthly telehealth vs in-person utilization and spending rates for mental health services among commercially insured US adults between 2019 and 2022.

## Methods

This cohort study quantified trends in mental health service utilization and spending in 3 periods: before the PHE declaration (January 1, 2019, to March 12, 2020), during the acute phase before vaccine availability (March 13, 2020, to December 17, 2020), and during the postacute phase (December 18, 2020, to August 31, 2022). We measured trends as the number of monthly medical claims per 1000 beneficiaries and spending per 10 000 beneficiaries among approximately 7 million commercially insured adults (aged ≥18 years). Claims were from self-insured employers offering Castlight Health as a health benefit,^4^ according to *International Statistical Classification of Diseases and Related Health Problems, Tenth Revision* diagnosis codes for anxiety disorders, major depressive disorder, bipolar disorder, schizophrenia, and posttraumatic stress disorder (eAppendix in Supplement 1).^5^ The RAND Institutional Review Board deemed this study exempt and waived informed consent because deidentified claims data were used. Analyses complied with the STROBE reporting guideline.

For each condition, we estimated a separate longitudinal, fixed-effects segmented regression for the 3 periods. Fixed effects were included for each month, state, and sex. Standard errors were clustered at the state level. Precision estimates are reported using 2-sided 95% CIs. Analysis was completed in March and May 2023 using Stata, version 16.0 (StataCorp).

## Results

We included data for 1 554 895 mental health service claims. During the acute phase, in-person visits decreased by 39.5% and telehealth visits increased roughly 10-fold (1019.3%) compared with the year prior (*P* < .001). Jointly, this represented a 22.3% increase in overall utilization (Table). These trends were generally consistent across conditions (Figure, A-C). During the postacute phase, telehealth visits stabilized at approximately 10 times (1068.3%) prepandemic levels, whereas in-person visits increased 2.2% each month over the period (both *P* = .002). By August 2022, in-person visits had returned to 79.9% of prepandemic levels; overall mental health service utilization was 38.8% higher than before the pandemic.

**Table.** Changes in In-Person and Telehealth Utilization and Spending for Mental Health Services Before and During the COVID-19 Pandemic (January 2019 to August 2022)^a^

Condition	Prepandemic				Acute phase				Postacute phase			
	In person		Telehealth		In person		Telehealth		In person		Telehealth	
	Coefficient (95% CI)	*P* value	Coefficient (95% CI)	*P* value	Coefficient (95% CI)	*P* value	Coefficient (95% CI)	*P* value	Coefficient (95% CI)	*P* value	Coefficient (95% CI)	*P* value
**Utilization**												
Depression (n = 507 951)												
Intercept	−11.49 (−34.01 to 11.04)	.31	−0.98 (−23.36 to 21.40)	.93	−1.70 (−2.01 to −1.40)	<.001	1.95 (1.48 to 2.42)	<.001	−1.66 (−1.93 to −1.39)	<.001	1.93 (1.48 to 2.38)	<.001
Slope	0.02 (−0.01 to 0.06)	.14	0.00 (−0.03 to 0.04)	.76	−0.05 (−0.07 to −0.03)	<.001	0.06 (0.04 to 0.08)	<.001	0.03 (0.01 to 0.05)	.002	−0.03 (−0.05 to −0.02)	<.001
Anxiety (n = 705 394)												
Intercept	−27.29 (−50.82 to −3.76)	.02	−18.38 (−41.19 to 4.40)	.11	−2.21 (−2.65 to −1.76)	<.001	2.92 (2.30 to 3.55)	<.001	−2.00 (−2.42 to −1.58)	<.001	3.13 (2.48 to 3.77)	<.001
Slope	0.05 (0.01 to 0.08)	.006	0.03 (−0.00 to 0.06)	.06	−0.05 (−0.07 to −0.02)	<.001	0.10 (0.07 to 0.13)	<.001	0.07 (0.05 to 0.09)	<.001	−0.03 (−0.04 to −0.01)	<.001
Bipolar disorder (n = 138 518)												
Intercept	13.10 (−3.96 to 30.16)	.13	8.20 (0.56 to 15.83)	.04	−0.43 (−0.56 to −0.30)	<.001	0.38 (0.19 to 0.57)	<.001	−0.45 (−0.58 to −0.32)	<.001	0.35 (0.16 to 0.54)	<.001
Slope	−0.01 (−0.04 to 0.01)	.23	−0.01 (−0.02 to 0.00)	.12	−0.01 (−0.02 to - 0.00)	<.001	0.01 (0.00 to 0.02)	.001	−0.00 (−0.01 to 0.00)	.38	−0.01 (−0.02 to −0.00)	.02
Schizophrenia (n = 41 688)												
Intercept	13.54 (5.10 to 21.98)	.002	7.39 (−0.19 to 14.97)	.06	−0.05 (−0.14 to 0.03)	.23	0.05 (−0.02 to 0.13)	.15	−0.02 (−0.24 to 0.20)	.86	−0.00 (−0.05 to 0.05)	.98
Slope	−0.02 (−0.03 to −0.00)	.009	−0.01 (−0.02 to 0.01)	.18	−0.00 (−0.02 to 0.01)	.73	0.00 (−0.00 to 0.00)	.74	−0.01 (−0.02 to 0.00)	.21	−0.00 (−0.01 to 0.00)	.03
PTSD (n = 160 804)												
Intercept	22.61 (−4.04 to 49.27)	.10	5.72 (−22.29 to 33.74)	.68	−0.54 (−0.73 to −0.34)	<.001	0.06 (−0.96 to 1.09)	.90	0.58 (−0.79 to −0.37)	<.001	0.07 (−0.97 to 1.11)	.89
Slope	−0.03 (−0.06 to 0.01)	.16	−0.00 (−0.04 to 0.03)	.84	−0.01 (−0.02 to 0.01)	.26	0.01 (0.00 to 0.02)	.02	0.01 (−0.01 to 0.02)	.34	0.01 (−0.02 to 0.00)	.10
Total (n = 1 554 895)												
Intercept	3.89 (−14.06 to 21.84)	.67	0.68 (8.16 to 9.51)	.88	−1.05 (−1.26 to −0.82)	<.001	1.09 (0.80 to 1.39)	<.001	−1.01 (−1.21 to −0.82)	<.001	1.10 (0.80 to 1.40)	<.001
Slope	0.00 (−0.02 to 0.03)	.95	0.00 (−0.01 to 0.02)	.62	−0.03 (−0.04 to −0.02)	<.001	0.04 (0.03 to 0.05)	<.001	0.02 (0.01 to 0.04)	.003	−0.02 (−0.03 to −0.01)	.002
**Spending**												
Depression (n = 507 951)												
Intercept	−89 001.48 (−130 094.60 to −47 908.33)	<.001	−15 102.41 (−60 890.30 to 30 685.48)	.51	−4302.16 (−5116.10 to −3488.22)	<.001	4882.77 (3785.87 to 5979.67)	<.001	−4784.64 (−5883.05 to −3686.23)	<.001	4760.49 (3642.13 to 5878.85)	<.001
Slope	145.30 (88.02 to 202.50)	<.001	27.56 (−36.26 to 91.37)	.39	−28.95 (−113.10 to 55.17)	.49	163.70 (120.90 to 206.50)	<.001	88.12 (56.38 to 119.90)	<.001	−93.73 (−125.40 to −62.04)	<.001
Anxiety (n = 705 934)												
Intercept	−74 391.30 (−117 231.50 to −31 551.13)	.001	−95 714.71 (−156 829.00 to −34 600.41)	.003	−5012.16 (−6032.08 to −3992.24)	<.001	7014.01 (5332.14 to 8696.88)	<.001	−5124.13 (−6464.30 to −3783.96)	<.001	7331.00 (5548.43 to 9113.57)	<.001
Slope	126.80 (67.11 to 186.50)	<.001	145.90 (60.98 to 230.90)	.001	14.49 (−95.78 to 124.80)	.79	314.50 (212.20 to 416.90)	<.001	160.70 (120.60 to 200.80)	<.001	−119.80 (−160.20 to −79.36)	<.001
Bipolar disorder (n = 138 518)												
Intercept	−11 430.45 (−35 771.08 to 12 910.18)	.35	−5173.84 (−25 758.95 to 15 411.27)	.62	−499.30 (−1071.08 to 72.42)	.09	967.90 (625.40 to 1310.00)	<.001	−424.10 (−1109.04 to 260.90)	.22	902.70 (555.50 to 1250.00)	<.001
Slope	23.48 (−10.57 to 57.52)	.17	10.75 (−17.98 to 39.48)	.46	−65.30 (−143.70 to 13.05)	.10	32.80 (6.12 to 59.48)	.02	2.53 (−12.73 to 17.79)	.74	−18.16 (−34.18 to −2.13)	.03
Schizophrenia (n = 41 688)												
Intercept	6218.23 (−674.10 to 13 110.59)	.08	2674.95 (185.20 to 5535.08)	.07	−286.40 (−599.10 to 26.22)	.07	119.80 (36.99 to 202.60)	.005	−487.90 (−2161.58 to 1185.78)	.56	148.20 (30.17 to 266.20)	.02
Slope	−7.54 (−17.08 to 2.00)	.12	−3.28 (−7.30 to 0.73)	.11	103.20 (−68.89 to 276.30)	.24	−4.78 (−16.44 to 6.89)	.42	−64.71 (−161.10 to 31.66)	.18	−4.84 (−8.15 to −0.82)	.02
PTSD (n = 160 804)												
Intercept	−17 297.09 (−41 627.16 to 7032.98)	.16	−69 289.94 (−127 045.40 to −11 534.45)	.02	−1282.85 (−1748.62 to −817.10)	<.001	1484.91 (551.30 to 2418.55)	.002	−1132.45 (−1577.76 to −687.10)	<.001	1354.02 (259.04 to 2449.00)	.02
Slope	39.05 (5.49 to 72.61)	.02	102.60 (22.15 to 183.10)	.01	−39.19 (−72.62 to −5.76)	.03	99.76 (50.12 to 149.40)	<.001	54.23 (32.13 to 76.33)	<.001	−36.91 (−60.24 to −13.58)	.003
Total (n = 1 554 895)												
Intercept	−35 759.00 (−58 075.05 to −13 442.95)	<.001	−31 014.44 (−56 384.07 to −5645.81)	.02	−2382.52 (−2825.07 to −1939.98)	<.001	2990.94 (2324.21 to 3657.67)	<.001	−2505.05 (−3009.51 to −2000.60)	<.001	2911.71 (2223.96 to 3599.46)	<.001
Slope	65.56 (34.59 to 96.53)	<.001	50.91 (15.71 to 86.10)	.005	−11.13 (−55.63 to 33.37)	.62	137.90 (102.10 to 173.70)	<.001	58.10 (36.59 to 79.60)	<.001	−57.27 (−75.47 to −39.07)	<.001

Abbreviation: PTSD, posttraumatic stress disorder.

^a^ Each column and row combination is a separate regression model. The regression model also includes controls for state, month, and sex of the patient. Standard errors were clustered at the state level.

**Figure.**
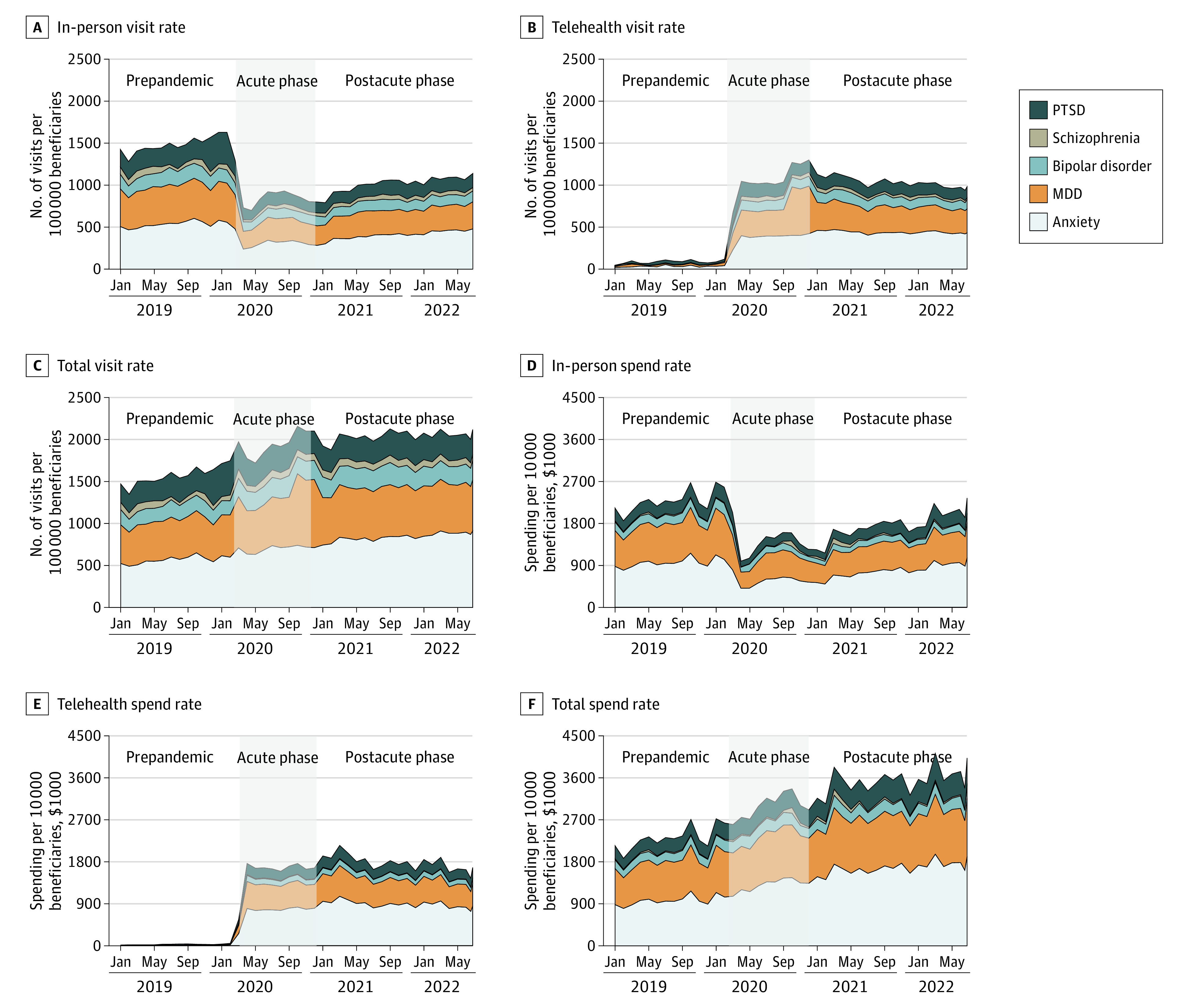
In-Person, Telehealth, and Total Mental Health Care Visits and Spending Before and During the COVID-19 Pandemic (January 2019 to August 2022) A to F, Visit and spending rates for in-person (A and D), telehealth (B and E), and total (C and F) services for mental health. MDD indicates major depressive disorder; PTSD, posttraumatic stress disorder.

Spending rates for mental health services mimicked utilization. During the acute phase, per capita expenditures were 29.5% higher (*P* < .001) compared with the year prior (Figure, D-F). During the postacute phase, there was a gradual increase in spending rates: spending for telehealth services remained stable, whereas spending for in-person care decreased to prepandemic levels. The average spending rate was $3 547 424 vs $2 308 247 per 10 000 beneficiaries per month in the postacute phase vs the prepandemic phase (a 53.7% increase).

## Discussion

In this cohort study, utilization and spending rates for mental health care services among commercially insured adults increased by 38.8% and 53.7%, respectively, between 2019 and 2022. This disproportionate increase in spending will likely evolve now that the PHE has ended, with insurers either continuing or stopping coverage for telehealth visits for mental health services.

This study has some limitations. First, the data represent approximately 7 million adults with employer-based private insurance. Utilization patterns, care needs, and spending may differ for other populations. Second, we were unable to distinguish new patients from existing patients receiving ongoing care. Finally, we were unable to examine trends by practitioner characteristics (eg, primary vs specialty care).

These findings suggest that telehealth utilization for mental health services remains persistent and elevated. If this increased utilization affects spending, insurers may begin rejecting the new status quo.^6^ This concern is particularly relevant when considered against the backdrop of telehealth policies that expired alongside the national PHE declaration.

## Notes

Supplement 1. **eAppendix.** Description of Data Sample and Procedures

Supplement 2.
Data Sharing Statement
