# Supplementary material for: Telehealth and In-Person Mental Health Service Utilization and Spending, 2019 to 2022
Source: JAMA Health Forum. 2023 Aug 25;4(8):e232645. doi: 10.1001/jamahealthforum.2023.2645 (PMC10457709; doi:10.1001/jamahealthforum.2023.2645)
Supplement: Supplement 1. — eAppendix. Description of Data Sample and Procedures [file jamahealthforum-e232645-s001.pdf]

Supplemental Online Content

Cantor JH, McBain RK, Ho PC, Bravata DM, Whaley C. Telehealth and in-person mental health service utilization and spending, 2019 to 2022. *JAMA Health Forum*. 2023;4(8):e232645. doi:10.1001/jamahealthforum.2023.2645

eAppendix. Description of Data Sample and Procedures

This supplemental material has been provided by the authors to give readers additional information about their work.

© 2023 Cantor JH et al. *JAMA Health Forum*.

eAppendix. Description of Data Sample and Procedures

This study used medical claims data from employers who purchased access to the Castlight Health platform. The platform provides price transparency, wellness and other health benefits tools. This analysis was restricted to the claims data that participating employers provide to Castlight as a way to implement the digital tools. For the approximately 137 self-insured employers that provide access to this tool, the healthcare claims data covers all in-network procedures that are reimbursed through insurance. All enrollees in the Castlight Health platform have behavioral health benefits. The data has reimbursement amounts, procedure codes, and patient diagnoses. **For this analysis our research team aggregated individual-level data to the condition, service setting (in-person, or telehealth), age, gender, state and month-level.** All reimbursement amounts were adjusted for inflation.

Definition of procedures
We used primary diagnosis codes for mental disorders at the 3- and 4-digit level from the International Statistical Classification of Diseases and Related Health Problems, Tenth Revision (ICD-10). The codes were for: major depressive disorder (F32, F33), anxiety disorders (F40, F41), bipolar disorder (F31), adjustment disorder (F43.2), and PTSD (F43.1)

In order to identify telemedicine utilization we used the following set of procedure codes: ('99441','99442','99443','99444','99421','99422','99423','98970','98971','98972','G2061','G2062','G2063'), claims with a procedure code modifier in ('95','GT','GQ'), or a place of service code equal to 2.

© 2023 Cantor JH et al. *JAMA Health Forum*.
