# Supplementary material for: Telehealth and In-Person Mental Health Service Utilization and Spending, 2019 to 2022
Source: JAMA Health Forum. 2023 Aug 25;4(8):e232645. doi: 10.1001/jamahealthforum.2023.2645 (PMC10457709; doi:10.1001/jamahealthforum.2023.2645)
Supplement: Supplement 2. — Data Sharing Statement [file jamahealthforum-e232645-s002.pdf]

# Data Sharing Statement

Cantor. Telehealth and In-Person Mental Health Service Utilization and Spending, 2019 to 2022. *JAMA Health Forum*. Published August 25, 2023.
doi:10.1001/jamahealthforum.2023.2645

**Data**
**Data available:** No

**Additional Information**
**Explanation for why data not available:** The data are from a private company.
